# The Global, Regional, and National Burden of Tracheal, Bronchus, and Lung Cancer Caused by Smoking: An Analysis Based on the Global Burden of Disease Study 2021

**DOI:** 10.5334/aogh.4572

**Published:** 2024-12-05

**Authors:** Jingting Zhang, Jincheng Tang, Renyi Yang, Siqin Chen, Huiying Jian, Puhua Zeng

**Affiliations:** 1 Hunan University of Chinese Medicine, Changsha, Hunan, China; 2 Hunan Provincial Hospital of Integrated Traditional Chinese and Western Medicine, Hunan Academy of Chinese Medicine, Changsha, Hunan, China

**Keywords:** Tracheal, bronchus, and lung cancer, smoking, Global Burden of Disease Study (GBD) 2021, mortality, disability‑adjusted life‑years (DALYs)

## Abstract

*Background:* Smoking is the primary risk factor for tracheal, bronchus, and lung (TBL) cancer.

*Objective:* This study aims to explore the epidemiological trends of smoking-attributable TBL cancer from 1990 to 2021.

*Methods:* Mortality and disability-adjusted life-years (DALYs) data for smoking-related TBL cancer from 1990 to 2021 were sourced from the Global Burden of Disease Study (GBD) 2021. Estimated annual percentage changes (EAPCs) were calculated to evaluate trends in age-standardized mortality rates (ASMRs) and age-standardized DALY rates (ASDRs). Additionally, the relationship between disease burden, EAPCs, and the sociodemographic index (SDI) was assessed.

*Findings:* Compared with 1990, both the mortality and DALYs due to smoking-related TBL cancer substantially increased by 2021. However, during this period, ASMR [EAPC: −0.97; 95% confidence interval (CI): −1.05 to −0.89] and ASDR (EAPC: −1.29; 95% CI: −1.37 to −1.22) demonstrated a downward trend. ASMR and ASDR in females were consistently lower than in males. In 2021, East Asia had the highest ASMR, while Central Europe recorded the highest ASDR, with Greenland exhibiting the highest ASMR and ASDR at the national level. Nationally, ASMR for smoking-related TBL cancer in 2021 showed a positive correlation with SDI, while the EAPC of both ASMR and ASDR from 1990 to 2021 displayed a negative correlation with SDI. Furthermore, in 2021, the greatest number of deaths from smoking-related TBL cancer occurred in individuals aged 70–74, while DALYs were highest in the 65–69 age group.

*Conclusions:* The burden of smoking-related TBL cancer varies across age, sex, geography, and SDI regions. Tailored public health interventions aligned with these epidemiological characteristics are essential for alleviating the disease burden.

## Introduction

Tracheal, bronchus, and lung (TBL) cancer represent a significant global public health challenge. In 2022, lung cancer accounted for 12.4% of all global cancer cases and 18.7% of cancer‑related deaths, ranking first in both categories [1]. Clinically, the majority of patients are diagnosed with lung cancer at an advanced stage, with an overall 5‑year survival rate of only about 20% [2]. In 2017, the financial burden of lung cancer in China was estimated at 25.069 billion US dollars (USD), and it is projected that by 2030, this economic burden will rise to 53.4 billion USD, requiring greater social resources [3]. Smoking is the primary risk factor for lung cancer, and the variation in lung cancer incidence largely reflects differences in smoking patterns. In some countries, tobacco control policies have effectively reduced lung cancer incidence [4, 5]. Cohort studies have shown that the risk of lung cancer incidence and mortality is significantly lower in non‑smokers compared with smokers [6]. Therefore, systematically understanding the epidemiological trends of smoking‑induced TBL cancer can help policymakers develop targeted strategies. While previous studies have analyzed lung cancer attributable to ambient fine particulate matter pollution and historical global epidemiological patterns of TBL cancer [7–9], there remains a lack of updated global data on smoking‑related TBL cancer.

The Global Burden of Disease (GBD) Study 2021 includes data on the incidence, mortality, and disability‑adjusted life‑years (DALYs) for 371 diseases and injuries across 204 countries and 811 regions from 1990 to 2021, along with 88 associated risk factors [10, 11]. In 2021, the risk factors for TBL cancer included environmental, behavioral, and metabolic factors, with smoking accounting for 58.89% of age‑standardized lung cancer mortality, surpassing the combined contribution of other risk factors (Supplementary Figure 1). This study, based on GBD 2021 data, analyzes the burden of smoking‑induced TBL cancer by gender, age, region, country, and sociodemographic index (SDI), aiming to provide insights for developing effective prevention and control strategies.

## Methods

### Data sources

All data were downloaded from the Global Health Data Exchange (GHDx) query tool (https://ghdx.healthdata.org/gbd-2021). The detailed methodology for the GBD 2021 estimation process has been previously described in the literature [10–12]. We used “deaths” and “DALYs” as the measures, “TBL cancer” as the cause, and “smoking” as the risk factor. The global burden of the disease was explored across 21 regions, 5 SDI regions, and 204 countries. This study utilized de‑identified publicly available data and adhered to the Guidelines for Accurate and Transparent Health Estimates Reporting [13].

### Definitions

The GBD 2021 study defines diseases using the International Classification of Disease (ICD) codes, specifically ICD‑9 and ICD‑10. In this study, TBL cancer is classified under ICD‑9 codes 162–162.9, 212.2–212.3, 231.1–231.2, 235.7, and ICD‑10 codes C33–C34.9, D02.1–D02.3, D14.2–D14.3, D38.1. Smoking is categorized under behavioral risks as a subcategory of tobacco, defined as the current or former active use of any tobacco product (excluding chewing tobacco and passive secondhand smoke). Exposure is measured by the number of cigarettes smoked per day and cumulative years of smoking [11, 14].

“Deaths” refers to the number of disease‑related deaths within a specific period. “DALYs” is the sum of years lived with disability (YLDs) and years of life lost (YLLs). YLDs represent the number of cases weighted by the severity of non‑fatal disabilities for a cause–age–sex–location–year‑specific population, while YLLs are calculated by multiplying the standard life expectancy at the time of death by the number of cause–age–sex–location–year‑specific deaths [10]. Both deaths and DALYs are critical measures for evaluating disease burden.

GBD 2021 divides the world into 21 regions based on geographic differences, including Central Asia, Central Sub‑Saharan Africa, Central Latin America, Oceania, and Western Europe, among others, and incorporates data from 204 countries and territories. Age groups are categorized into 20 groups, spanning from ages <5 years to ≥95 years, with each group representing a 5‑year increment. The sociodemographic index (SDI) quantifies the socioeconomic development of countries and regions, and it is closely linked to health outcomes. The SDI ranges from 0 to 1, with lower values indicating poorer urban development and higher values representing greater socioeconomic advancement. Based on lag distributed income per capita, total fertility rate under age 25, and average years of education for those aged 15 and above, 204 countries and regions are classified into low, low‑middle, middle, high‑middle, and high SDI categories [15].

### Statistical analysis

The GBD Cause of Death Ensemble modeling (CODEm) was used to generate mortality estimates across different genders, ages, time periods, and regions [16]. Based on GBD 2021 global population data, we quantified the burden of smoking‑related TBL cancer in different regions using age‑standardized mortality rates (ASMRs) and age‑standardized DALY rates (ASDRs) to eliminate biases arising from population and age composition differences. For deaths, DALYs, ASMRs, and ASDRs, the 95% uncertainty interval (UI) was derived from the 25th and 975th values of 1000 draws from the uncertainty distribution.

We calculated the estimated annual percentage changes (EAPCs) and their corresponding 95% confidence intervals (CIs) using linear regression models to describe trends in age‑standardized rates (ASRs) of smoking‑related TBL cancer from 1990 to 2021. EAPC values with 95% CIs greater than 0 indicate an increasing trend in ASRs, while those less than 0 indicate a decreasing trend. The methods for calculating ASR and EAPC have been previously described in the literature [17, 18]. Additionally, we used the Spearman rank test to examine the association between smoking‑related TBL cancer burden, EAPCs, and SDI [19, 20]. All analyses and visualizations were conducted using Software R (version 4.2.2) and R Studio, with a two‑tailed *P* value of less than 0.05 considered statistically significant.

## Results

### Global deaths and DALYs

Globally, in 2021, the number of smoking‑attributable TBL cancer deaths was 1,195,795.65, representing a significant increase of 62.05% compared with 1990. The ASMR decreased from 18.72/100,000 in 1990 to 13.85/100,000 in 2021. Additionally, the EAPC for ASMR from 1990 to 2021 was −0.97, leading to an overall decline in ASMR of 26.01%. In 2021, female smoking‑attributable TBL cancer deaths numbered 220,125.05, 77.44% fewer than in males, indicating a significant gender disparity. However, the percentage increase in deaths among females between 1990 and 2021 [88.03% versus 57.15%] and the EAPC for ASMR were higher than for males. Notably, female ASMR showed no significant change from 1990 to 2005, only beginning to decline slowly after 2005. From 1990 to 2021, while the number of smoking‑attributable TBL cancer deaths increased, ASMR showed a downward trend, and both deaths and ASMR in females remained significantly lower than in males (Table 1, Supplementary Figure 2A).

**Table 1:** Global deaths and DALYs of smoking‑induced tracheal, bronchus, and lung cancer from 1990 to 2021.

YEAR	BOTH	FEMALE	MALE
1990			
Deaths (95% UI)	737,925.68 (686,229.20–791,982.05)	117,068.52 (105,907.20–128,707.03)	620,857.17 (577,879.96–669,544.06)
DALYs (95% UI)	19,356,963.13 (18,071,732.80–20,777,679.76)	2,873,527.83 (2,617,006.43–3,137,842.37)	16,483,435.30 (15,356,336.71–17,770,216.31)
ASMR/100,000 persons (95% UI)	18.72 (17.37–20.12)	5.55 (5.00–6.11)	34.85 (32.37–37.57)
ASDR/100,000 persons (95% UI)	471.15 (438.80–505.43)	133.69 (121.53–145.86)	854.09 (796.48–920.94)
2021			
Deaths (95% UI)	1,195,795.65 (1,054,669.94–1,359,222.56)	220,125.05 (187,188.66–255,058.71)	975,670.59 (843,775.90–1,123,054.48)
DALYs (95% UI)	27,713,689.18 (24,404,593.21–31,596,581.23)	4,780,176.62 (4,165,305.62–5,493,258.44)	22,933,512.55 (19,793,991.37–26,526,587.66)
ASMR/100,000 persons (95% UI)	13.85 (12.22–15.74)	4.72 (4.02–5.47)	24.68 (21.38–28.37)
ASDR/100,000 persons (95% UI)	315.67 (278.19–359.86)	103.13 (89.95–118.38)	553.82 (479.27–639.64)
1990‑2021			
PC of deaths (%)	62.05 (39.75–85.51)	88.03 (68.70–108.75)	57.15 (31.66–83.99)
PC of DALYs (%)	43.17 (23.41–64.74)	66.35 (50.21–83.37)	39.13 (15.84–63.97)
EAPC of ASMR (95% CI)	−0.97 (−1.05 to −0.89)	−0.59 (−0.75 to −0.43)	−1.10 (−1.16 to −1.03)
EAPC of ASDR (95% CI)	−1.29 (−1.37 to −1.22)	−0.86 (−1.02 to −0.70)	−1.39 (−1.45 to −1.33)

DALYs, disability‑adjusted life‑years; ASMR, age‑standardized mortality rate; ASDR, age‑standardized disability‑adjusted life‑year rate; PC, percentage change; EAPC, estimated annual percentage change; UI, uncertainty interval; CI, confidence interval.

Globally, the number of DALYs increased by 43.17%, rising from 19,356,963.13 in 1990 to 27,713,689.18 in 2021. However, ASDR decreased by 33%, from 471.15/100,000 in 1990 to 315.67/100,000 in 2021, with an EAPC of −1.29. Additionally, in 2021, the number of DALYs in males [22,933,512.55 versus 4,780,176.62] and ASDR [553.82/100,000 versus 103.13/100,000] were significantly higher than in females. However, the percentage increase in DALYs for males (39.13%) was much lower than for females (66.35%) between 1990 and 2021. Overall, the ASDR for smoking‑attributable TBL cancer significantly declined from 1990 to 2021, and the ASDR for females remained much lower than for males (Table 1, Supplementary Figure 2B).

### Regional deaths and DALYs

**Figure 1:**
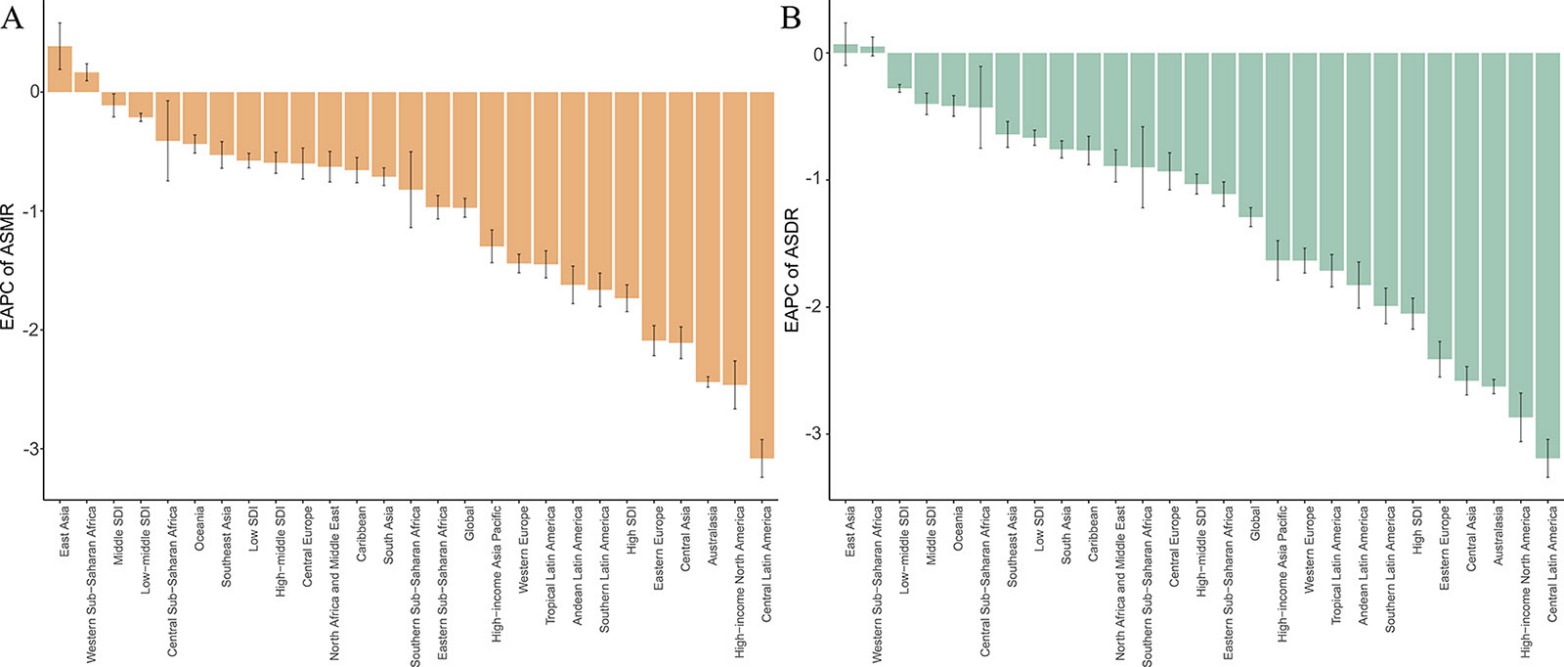
EAPCs of the ASRs for smoking‑induced TBL cancer from 1990 to 2021. **A**. EAPCs of the ASMR for smoking‑induced TBL cancer in 21 regions and 5 SDI regions. **B**. EAPCs of the ASDR for smoking‑induced TBL cancer in 21 regions and 5 SDI regions. ASRs, age‑standardized rates; ASMR, age‑standardized mortality rate; ASDR, age‑standardized disability‑adjusted life‑year rate; EAPC, estimated annual percentage change; SDI, sociodemographic index; TBL cancer, tracheal, bronchus, and lung cancer.

**Figure 2:**
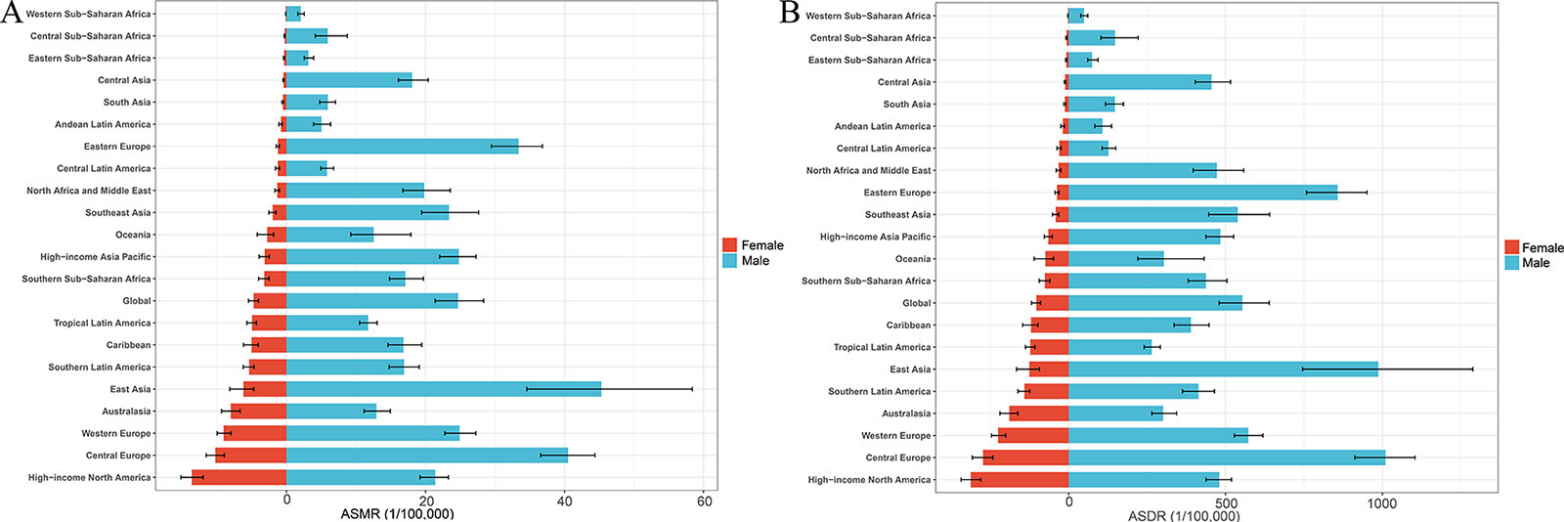
Gender differences in ASRs for smoking‑induced TBL cancer in 2021. **A**. Gender differences in ASMR for smoking‑induced TBL cancer across 21 regions. **B**. Gender differences in ASDR for smoking‑induced TBL cancer across 21 regions. ASRs, age‑standardized rates; ASMR, age‑standardized mortality rate; ASDR, age‑standardized disability‑adjusted life‑year rate; TBL cancer, tracheal, bronchus, and lung cancer.

At the regional level, in 2021, the top three regions with the highest number of smoking‑attributable TBL cancer deaths were East Asia (529,329.30), Western Europe (148,841.48), and high‑income North America (116,171.00). The region with the highest ASMR was East Asia (24.15/100,000). From 1990 to 2021, only two regions, East Asia (EAPC: 0.39) and Western Sub‑Saharan Africa (EAPC: 0.17), experienced a positive EAPC in ASMR, indicating an overall increasing trend in smoking‑related TBL cancer mortality rates, with the largest increase in East Asia. In contrast, the other 19 GBD regions showed a negative EAPC for ASMR, reflecting an overall decreasing trend. Central Latin America had the steepest decline in ASMR, with an EAPC of −3.08, followed by high‑income North America and Australasia. Additionally, in 2021, the ASMR for smoking‑related TBL cancer in men was significantly higher than in women across all regions, with the highest ASMR in men observed in East Asia and the highest in women in high‑income North America (Supplementary Table 1, Figures 1A and 2A).

Similarly, in 2021, the highest DALYs attributable to smoking‑related TBL cancer were observed in East Asia (12,198,631.52). Central Europe had the highest ASDR (608.12/100,000), followed by East Asia (538.21/100,000) and high‑income North America (390.40/100,000). From 1990 to 2021, East Asia showed the largest increase in ASDR (EAPC: 0.07). Except for East Asia and Western Sub‑Saharan Africa, all other regions exhibited a declining trend in ASDR. The largest decline occurred in Central Latin America (EAPC: −3.19). Notably, in 2021, the ASDR for smoking‑related TBL cancer in men was significantly higher than in women across all regions, with the highest male ASDR in Central Europe and the highest female ASDR in high‑income North America (Supplementary Table 1, Figures 1B and 2B).

### National deaths and DALYs

**Figure 3:**
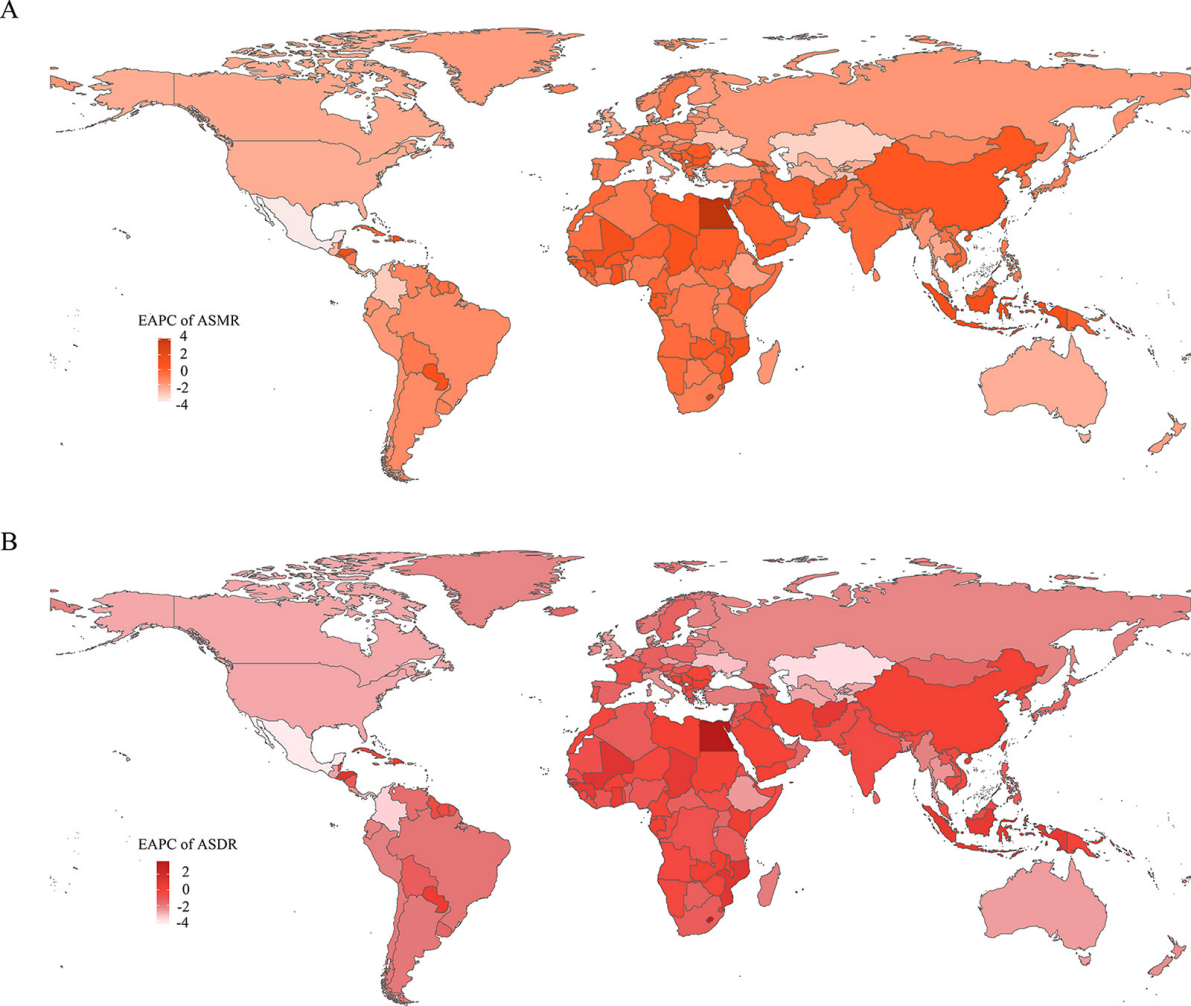
EAPCs of the ASRs for smoking‑induced TBL cancer from 1990 to 2021. **A**. EAPCs of the ASMR for smoking‑induced TBL cancer in 204 countries and regions. **B**. EAPCs of the ASDR for smoking‑induced TBL cancer in 204 countries and regions. ASRs, age‑standardized rates; ASMR, age‑standardized mortality rate; ASDR, age‑standardized disability‑adjusted life‑year rate; EAPC, estimated annual percentage change; TBL cancer, tracheal, bronchus, and lung cancer.

At the national level, in 2021, the countries with the highest number of smoking‑attributable TBL cancer deaths were China (518,796.30), followed by the United States of America (103,752.30) and Japan (49,851.35). The countries with the highest ASMRs were Greenland (43.76/100,000), Monaco (39.42/100,000), and Montenegro (34.29/100,000). Between 1990 and 2021, 46 countries experienced an overall increase in smoking‑related TBL cancer ASMR, while 157 countries saw a decrease. The largest increase in ASMR was observed in Egypt (EAPC: 4.15), Lesotho (EAPC: 2.97), and Georgia (EAPC: 1.49). Conversely, the countries with the largest decreases in ASMR were Mexico (EAPC: −4.27), Kazakhstan (EAPC: −3.49), and Singapore (EAPC: −3.35). Notably, the ASMR for smoking‑related TBL cancer in the Democratic People’s Republic of Korea remained stable, with no significant upward or downward trend (Supplementary Table 2, Figure 3A).

In 2021, the countries with the highest smoking‑related TBL cancer DALYs were China (11,942,343.33), the United States of America (2,274,968.50), and the Russian Federation (931,684.96). Similar to ASMR, the countries with the highest ASDRs were Greenland (1,066.51/100,000), Monaco (972.73/100,000), and Montenegro (889.64/100,000). Between 1990 and 2021, ASDRs increased in 36 countries and decreased in 166. Egypt had the largest increase in ASDR (EAPC: 3.65), followed by Lesotho (EAPC: 3.25) and Guinea‑Bissau (EAPC: 1.33). In contrast, the largest decreases in ASDR were in Mexico (EAPC: −4.39), Kazakhstan (EAPC: −3.85), and Singapore (EAPC: −3.70). It is noteworthy that the ASDR for smoking‑related TBL cancer in the Democratic People’s Republic of Korea and Timor‑Leste remained stable, showing no significant change (Supplementary Table 2, Figure 3B).

### Burden of smoking‑induced TBL cancer based on SDI

At the SDI regional level, in 2021, the High‑middle SDI region recorded the highest smoking‑induced TBL cancer deaths (414,797.64) and DALYs (9,802,635.50). This was followed by the middle SDI and high SDI regions. Similarly, the high‑middle SDI region also had the highest ASMR (20.56/100,000) and ASDR (486.63/100,000). Additionally, from 1990 to 2021, ASMR and ASDR of smoking‑induced TBL cancer in all five SDI regions showed varying degrees of decline, with the high SDI region exhibiting the most significant decrease. The EAPC for ASMR and ASDR in this region was −1.73 and −2.05, respectively. Notably, the ASMR and ASDR for females in the high SDI region consistently remained higher than in the other four SDI regions, while males in the high‑middle SDI region also had higher ASMR and ASDR compared with other SDI regions. Furthermore, between 1990 and 2021, the decline in ASMR and ASDR for males in both the high‑middle and high SDI regions was significantly greater than that for females (Supplementary Table 1, Supplementary Figure 2).

**Figure 4:**
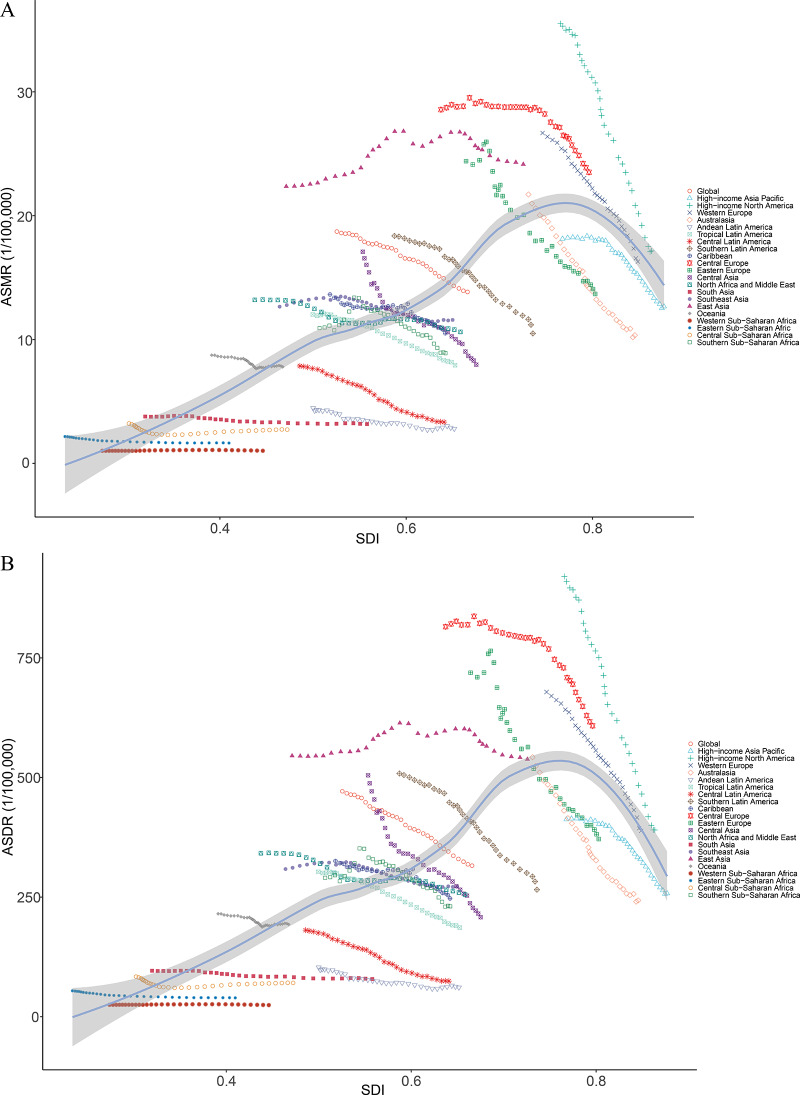
ASRs for smoking‑induced TBL cancer of 21 regions by SDI. **A**. ASMRs for smoking‑induced TBL cancer of 21 regions from 1990−2021 according to the SDI. **B**. ASDRs for smoking‑induced TBL cancer of 21 regions from 1990−2021 according to the SDI. ASRs, age‑standardized rates; ASMR, age‑standardized mortality rate; ASDR, age‑standardized disability‑adjusted life‑year rate; SDI, sociodemographic index; TBL cancer, tracheal, bronchus, and lung cancer.

**Figure 5:**
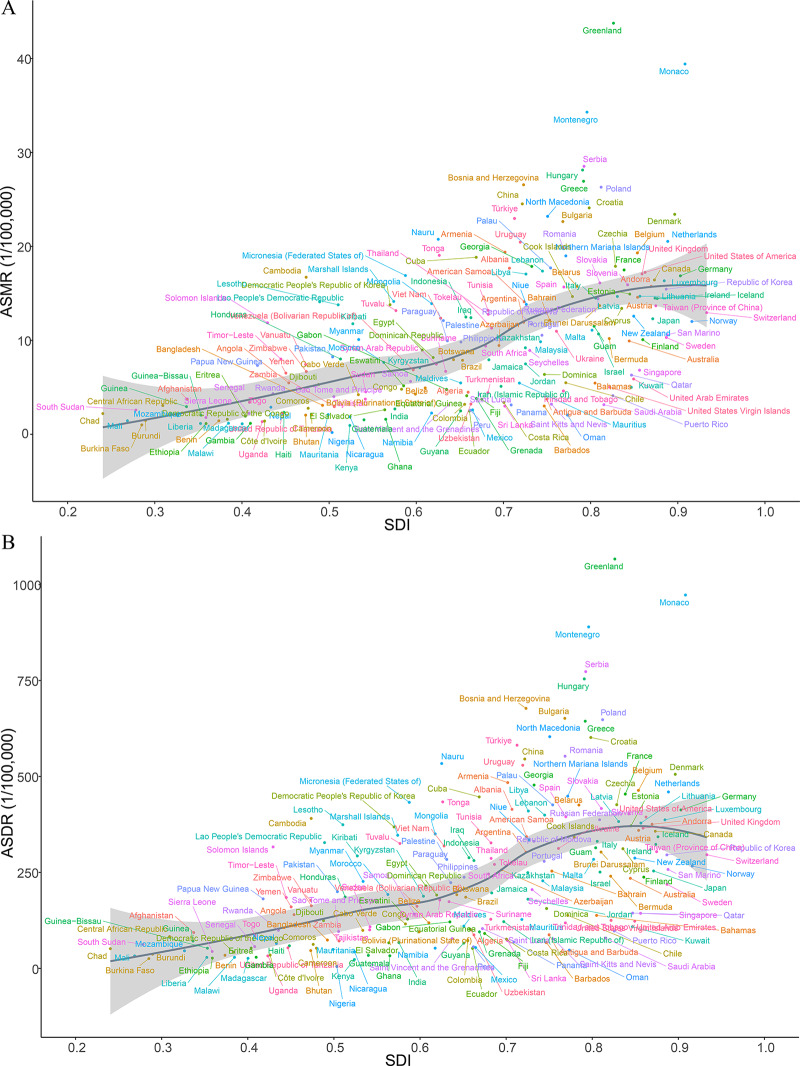
ASRs for smoking‑induced TBL cancer of 204 countries and territories by SDI. **A**. ASMRs for smoking‑induced TBL cancer of 204 countries and territories in 2021 according to the SDI. **B**. ASDRs for smoking‑induced TBL cancer of 204 countries and territories in 2021 according to the SDI. ASRs, age‑standardized rates; ASMR, age‑standardized mortality rate; ASDR, age‑standardized disability‑adjusted life‑year rate; SDI, sociodemographic index; TBL cancer, tracheal, bronchus, and lung cancer.

**Figure 6:**
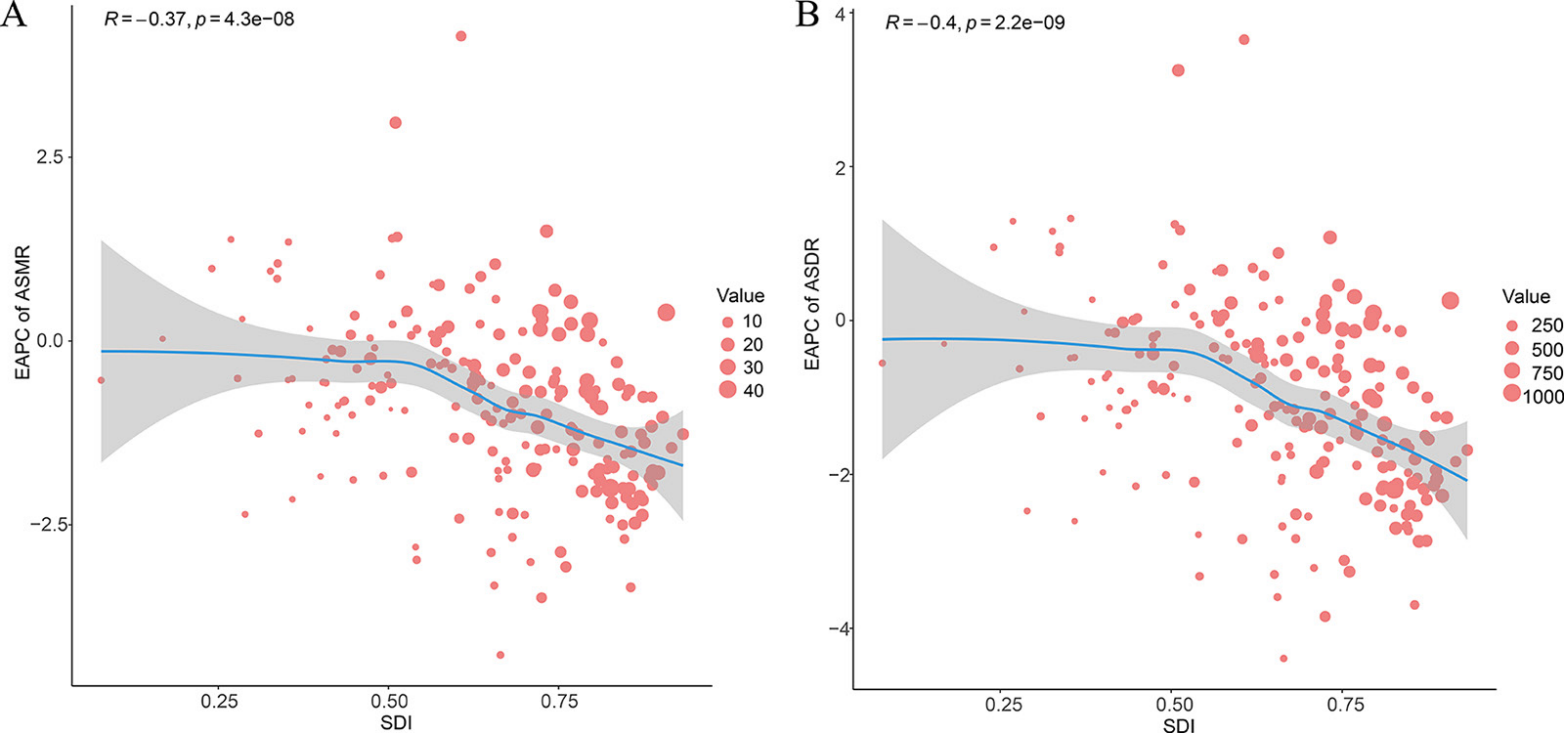
EAPCs of the ASRs for smoking‑induced TBL cancer in 204 countries and territories by SDI. **A**. EAPCs of the ASMRs for smoking‑induced TBL cancer in 204 countries and territories by SDI in 2021. **B**. EAPCs of the ASDRs for smoking‑induced TBL cancer in 204 countries and territories by SDI in 2021. ASRs, age‑standardized rates; ASMR, age‑standardized mortality rate; ASDR, age‑standardized disability‑adjusted life‑year rate; EAPC, estimated annual percentage change; SDI, sociodemographic index; TBL cancer, tracheal, bronchus, and lung cancer.

Between 1990 and 2021, a negative correlation was observed between ASMR and ASDR of smoking‑induced TBL cancer and SDI levels, except in East Asia, in regions with medium and high SDI levels. In regions with low SDI levels, changes in SDI did not significantly impact ASMR and ASDR. At the national level, in 2021, there was a positive correlation between smoking‑induced TBL cancer ASMR and SDI. ASDR exhibited a positive correlation with SDI at low and medium SDI levels, while in regions with high SDI levels, it gradually decreased with increasing SDI. Moreover, the ASMR and ASDR in low SDI regions were generally lower than in high SDI regions. From 1990 to 2021, there was a negative correlation between EAPC of ASMR and ASDR and SDI, a relationship that became more pronounced when SDI exceeded 0.5, with EAPC increasingly tending toward negative values as SDI increased (Figures 4, 5, 6).

### Burden of smoking‑induced TBL cancer based on age and sex

**Figure 7:**
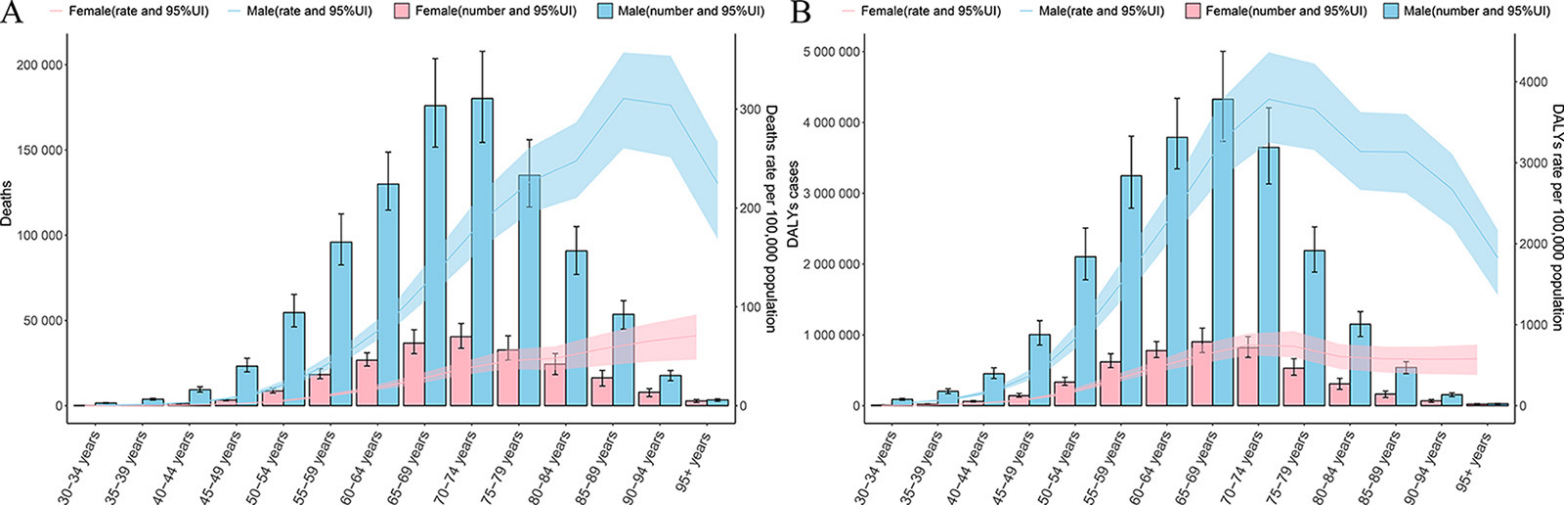
Age‑specific numbers and rates of deaths and DALYs of smoking‑induced TBL cancer by gender in 2021. **A**. Age‑specific numbers and rates of deaths from smoking‑induced TBL cancer. **B**. Age‑specific numbers and rates of DALYs from smoking‑induced TBL cancer. DALYs, disability‑adjusted life‑years; TBL cancer, tracheal, bronchus, and lung cancer.

In 2021, the highest number of smoking‑induced TBL cancer deaths occurred in both men and women aged 70–74. Before age 74, the number of deaths increased with age for both sexes, but after 74, the number of deaths decreased as age increased. There was a positive correlation between age‑specific mortality rate and age in women, while for men, the age‑specific mortality rate peaked at 85–89 years and then gradually declined. The majority of DALYs due to smoking‑induced TBL cancer were observed in the 65–69 age group. The age‑specific DALY rate for both men and women increased with age until 74, after which it showed a negative correlation with age. Additionally, the age‑specific DALY rate in women showed little variation after age 80. It is noteworthy that at all age groups, the age‑specific mortality rate and age‑specific DALY rate for men were higher than those for women, and no smoking‑induced TBL cancer deaths or DALYs were observed in individuals under 30 (Figure 7).

## Discussion

This study, utilizing the latest GBD 2021 data, analyzed the epidemiological trends of smoking‑induced TBL cancer at global, regional, and national levels, revealing significant changes in the disease burden compared to 1990. In 2021, 58.89% of TBL cancer deaths were attributable to smoking, far surpassing the contribution of other risk factors such as particulate matter pollution, occupational carcinogens, and high fasting plasma glucose. From 1990 to 2021, global deaths and DALYs attributable to smoking‑induced TBL cancer increased significantly, which may be associated with global population growth and the intensification of population aging [21]. The expansion of the global population tends to increase the absolute number of cancer cases and deaths, adding pressure on healthcare systems [22]. The growing elderly population is more prone to immune decline, further increasing the risk of cancer [23]. However, ASMR and ASDR have shown a declining trend, possibly owing to advancements in medical technology and the implementation of more robust tobacco control policies. Additionally, the signing of the WHO Framework Convention on Tobacco Control in 2003, along with the increased public media campaigns highlighting the harms of smoking, have contributed to reducing tobacco use and alleviating the global burden of smoking‑related TBL cancer [24, 25]. Smoking cessation after TBL cancer diagnosis also plays a crucial role in improving both overall and progression‑free survival. Recent studies have shown that smoking cessation at any stage of lung cancer can significantly reduce treatment complications, improve immune responses, and enhance the efficacy of chemotherapeutic and immunotherapeutic agents [26]. This underscores the critical role of cessation programs in the overall management of smoking‑induced TBL cancer.

The burden of smoking‑induced TBL cancer varies significantly across different regions and countries. From 1990 to 2021, ASMR and ASDR increased only in East Asia and Western Sub‑Saharan Africa, while declining in 19 other regions. This disparity may be attributed to differences in tobacco control measures and the timeline of smoking prevalence across regions. In contrast to regions like Europe and North America, East Asia’s early tobacco control efforts were insufficient, and many countries only implemented stringent policies in the last decade. Additionally, rapid economic development in the region has led to lifestyle changes, with smoking often regarded as a symbol of social status, contributing to the rise in smoking rates [27]. In Western Sub‑Saharan Africa, economic development over the past 30 years has expanded the tobacco market, with tobacco companies targeting low‑income populations with affordable products [28]. Public health policies remain relatively weak, and there is insufficient awareness of the dangers of smoking [29]. Combined with limited healthcare resources, where the focus is predominantly on maternal health and infectious diseases, cancer prevention and management are often neglected, leading to increasing ASMR and ASDR in this region [30]. Moreover, in some low‑income regions, cultural norms and economic dependence on tobacco farming complicate the implementation of comprehensive anti‑smoking campaigns, further exacerbating the public health challenge.

In contrast, regions such as Western Europe, North America, and Australia implemented effective tobacco control measures early on, imposed heavy taxes on tobacco, and benefited from abundant healthcare resources, resulting in a decline in ASMR and ASDR. Australia’s plain packaging legislation and strict tobacco advertising restrictions have significantly reduced smoking prevalence, serving as a global model for tobacco control policies [31]. Similarly, North America’s integration of public health initiatives with educational programs has strengthened the effectiveness of anti‑smoking campaigns [32].

In 2021, among the top 10 countries with the highest ASMR for smoking‑induced TBL cancer, China ranked 9th, while 8 of the top 10 were European countries, most of which were located in Eastern Europe or the Balkans. These European countries have a deep‑rooted smoking culture, where smoking remains socially acceptable, especially in social interactions [33]. Although tobacco control measures, such as restrictions on smoking advertisements and enhanced public smoking bans, have been implemented in recent years, these policies were introduced later and with less intensity compared to countries in North America and Western Europe [22, 34]. Furthermore, these countries face significant gaps in early cancer screening and diagnosis [35]. Tobacco taxes in these nations are relatively low, making smoking economically feasible. In China, despite strengthened tobacco control measures in recent years, enforcement remains relatively weak compared with other countries, particularly in rural areas, and there is an imbalance in the distribution of healthcare resources between urban and rural regions [36]. The large number of smokers over the past decades is now entering the high‑risk age for TBL cancer [37]. This highlights the urgent need for expanding healthcare infrastructure and ensuring equitable access to smoking cessation services and cancer screening programs in rural regions.

Most cancers exhibit significant sociodemographic disparities [38]. In 2021, the high‑middle SDI region had the highest ASMR and ASDR for smoking‑induced TBL cancer. A possible explanation is the historically high smoking rates in high‑middle SDI regions (such as parts of South America, Eastern Europe, and East Asia) during the mid to late 20th century, combined with the long latency period of smoking‑induced TBL cancer, which could have led to the current peak in ASMR and ASDR. Additionally, these regions often face significant wealth disparities, where lower‑income populations have greater access to tobacco and limited access to early disease screening and quality healthcare services [22, 39]. From 1990 to 2021, the high SDI region showed the most significant decline in ASMR and ASDR, attributed to the widespread establishment of smoking cessation services in many developed countries, offering counseling, pharmacotherapy, and online support [40]. There is also a focus on youth through school education and strict regulations on tobacco purchasing age and sales channels, limiting the influx of new smokers [41]. Furthermore, comprehensive cancer screening, prevention programs, and advancements in cancer treatment have further reduced the burden of smoking‑induced TBL cancer in these regions [42].

In 2021, deaths and DALYs from smoking‑induced TBL cancer were predominantly concentrated among middle‑aged and elderly populations. This is likely due to the cumulative nature of lung mutations and DNA damage caused by smoking, which reach their peak in middle to older age. Additionally, the immune system and repair mechanisms in older individuals tend to weaken, often accompanied by other chronic conditions [43]. Many patients do not undergo health screenings until they retire in middle or old age, by which point the disease has often progressed to a late stage, with more rapid malignant development [44]. Furthermore, the ASMR and ASDR for smoking‑induced TBL cancer have consistently been higher in men than in women, which could be attributed to the historically higher smoking rates among men. Smoking among men is more socially accepted, while women face more societal restrictions regarding smoking [45]. However, in certain regions, rising smoking rates among women and young adults necessitate targeted prevention strategies, particularly through culturally sensitive public health campaigns.

The implementation of lung cancer screening programs, particularly those utilizing low‑dose computed tomography (LDCT), has shown promise in reducing mortality rates by facilitating early detection [46]. For instance, the United States of America initiated the National Lung Screening Trial (NLST), which demonstrated a 20% reduction in lung cancer mortality among high‑risk populations undergoing LDCT screening compared with chest radiography. Similarly, European countries have launched pilot programs to assess the feasibility and effectiveness of LDCT screening [47]. However, the adoption of such programs varies globally, with low‑ and middle‑income countries facing challenges related to resource allocation, infrastructure, and accessibility.

This study provides an updated global and regional burden analysis of smoking‑induced TBL cancer. It examines epidemiological trends based on age, gender, geography, and SDI levels. The findings offer valuable insights for governments and public health organizations to implement more targeted policies and interventions, such as strengthening anti‑smoking legislation (e.g., higher tobacco taxes and stricter advertising restrictions), increasing public awareness campaigns about the dangers of smoking, and expanding access to smoking cessation programs [48]. In addition, prioritizing equitable access to healthcare services, particularly in low‑ and middle‑income countries, and fostering international collaborations to share successful tobacco control models could further alleviate the burden of smoking‑induced TBL cancer globally. These measures can help optimize the allocation of healthcare resources, promote regional collaboration and assistance, and ultimately minimize the future burden of smoking‑related TBL cancer.

This study has certain limitations. First, it relies on public health data collected by GBD 2021 from various countries, and in some regions, the data collection may be incomplete or inaccurate due to inadequacies in healthcare systems, potentially introducing bias in the burden analysis. Second, GBD data does not cover all regions and racial groups globally, summarizing only specific areas. Third, changes in smoking exposure will have a delayed effect on TBL cancer incidence, and therefore, there will be a lag before any recently introduced interventions, such as tobacco control legislation, affect outcomes. The medium‑ to long‑term success of these policies may not yet be reflected in the current burden data. Finally, smoking‑related data often come from subjective patient reports, which may lead to an underestimation or overestimation of the disease burden.

## Conclusions

Despite the decline in the burden of smoking‑induced TBL cancer over the past 30 years, it remains a significant global health risk and challenge. Therefore, it is essential to implement more targeted tobacco control policies and public health interventions based on the geographical, age, and population distribution of the disease. Efforts should focus on enhancing public health education campaigns, raising awareness of early cancer screening, and strengthening early detection practices among the population to mitigate the future societal impact of smoking‑induced TBL cancer.

## Data Availability

The validation dataset was available on request. Further information can be directed to the corresponding author. The data used for these analyses are all publicly available at https://ghdx.healthdata.org/gbd-2021.

## Additional Files

The additional files for this article can be found as follows:

Supplementary File 1. Table 1. Deaths and disability-adjusted life years (DALYs) due to smoking‑induced tracheal, bronchial, and lung cancer across 21 regions and 5 sociodemographic index (SDI) regions from 1990 to 2021.

Supplementary File 2. Table 2. Deaths and disability-adjusted life years (DALYs) due to smoking‑induced tracheal, bronchial, and lung cancer across 204 countries and territories from 1990 to 2021.

Supplementary File 3. Figure S1. Proportion of attributable risk factors for deaths from tracheal, bronchus, and lung cancer globally in 2021.

Supplementary File 4. Figure S2. Trends in age‑standardized mortality rate (ASMR) and age‑standardized disability-adjusted life years rate (ASDR) for tracheal, bronchus, and lung cancer globally by gender from 1990 to 2021.
