# Supplementary figures and images for: The Global, Regional, and National Burden of Tracheal, Bronchus, and Lung Cancer Caused by Smoking: An Analysis Based on the Global Burden of Disease Study 2021
Source: Ann Glob Health. 2024 Dec 5;90(1):77. doi: 10.5334/aogh.4572 (PMC11623080; doi:10.5334/aogh.4572)

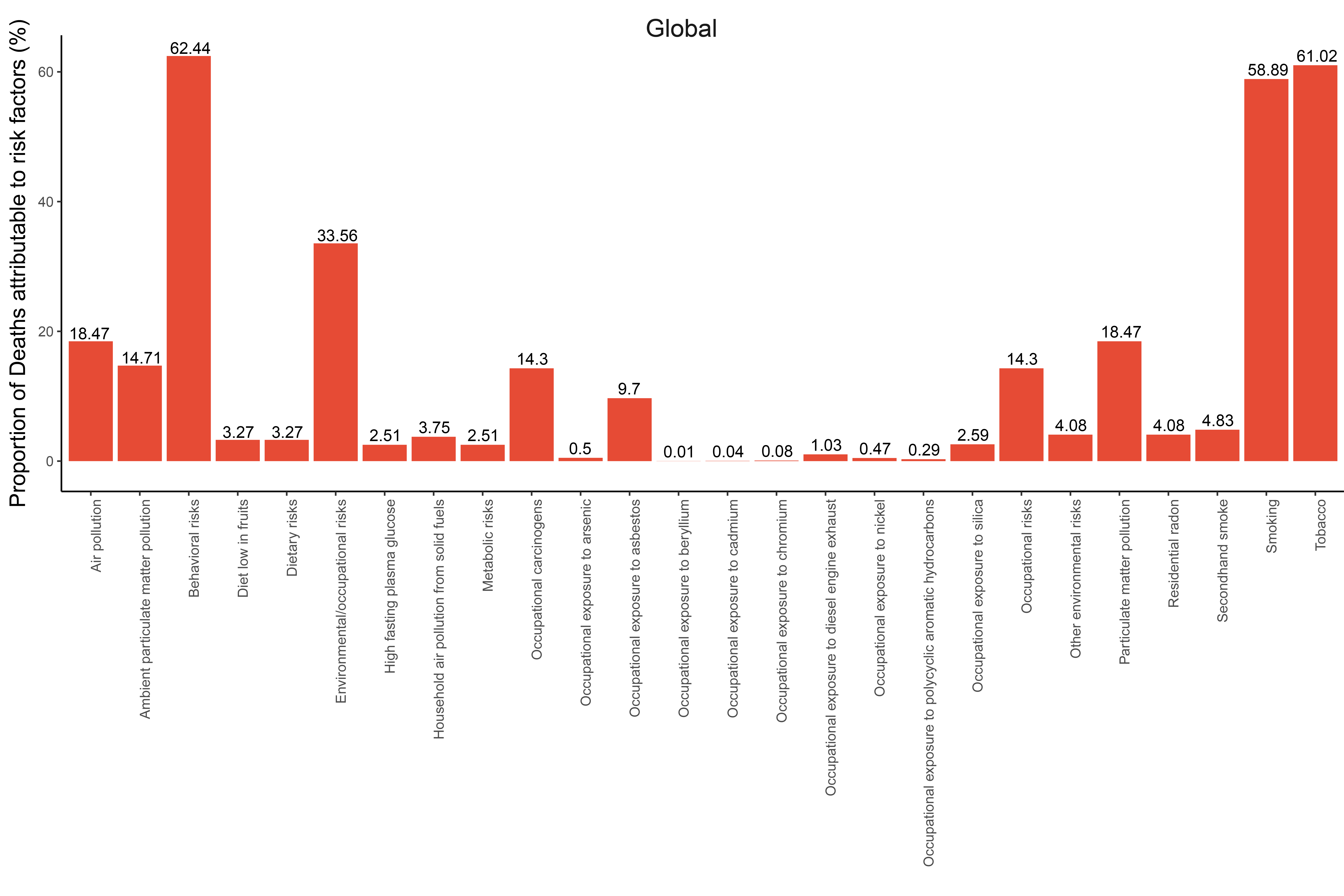

Supplement: Supplementary File 3. — Figure S1. Proportion of attributable risk factors for deaths from tracheal, bronchus, and lung cancer globally in 2021. [file agh-90-1-4572-s3.tiff]

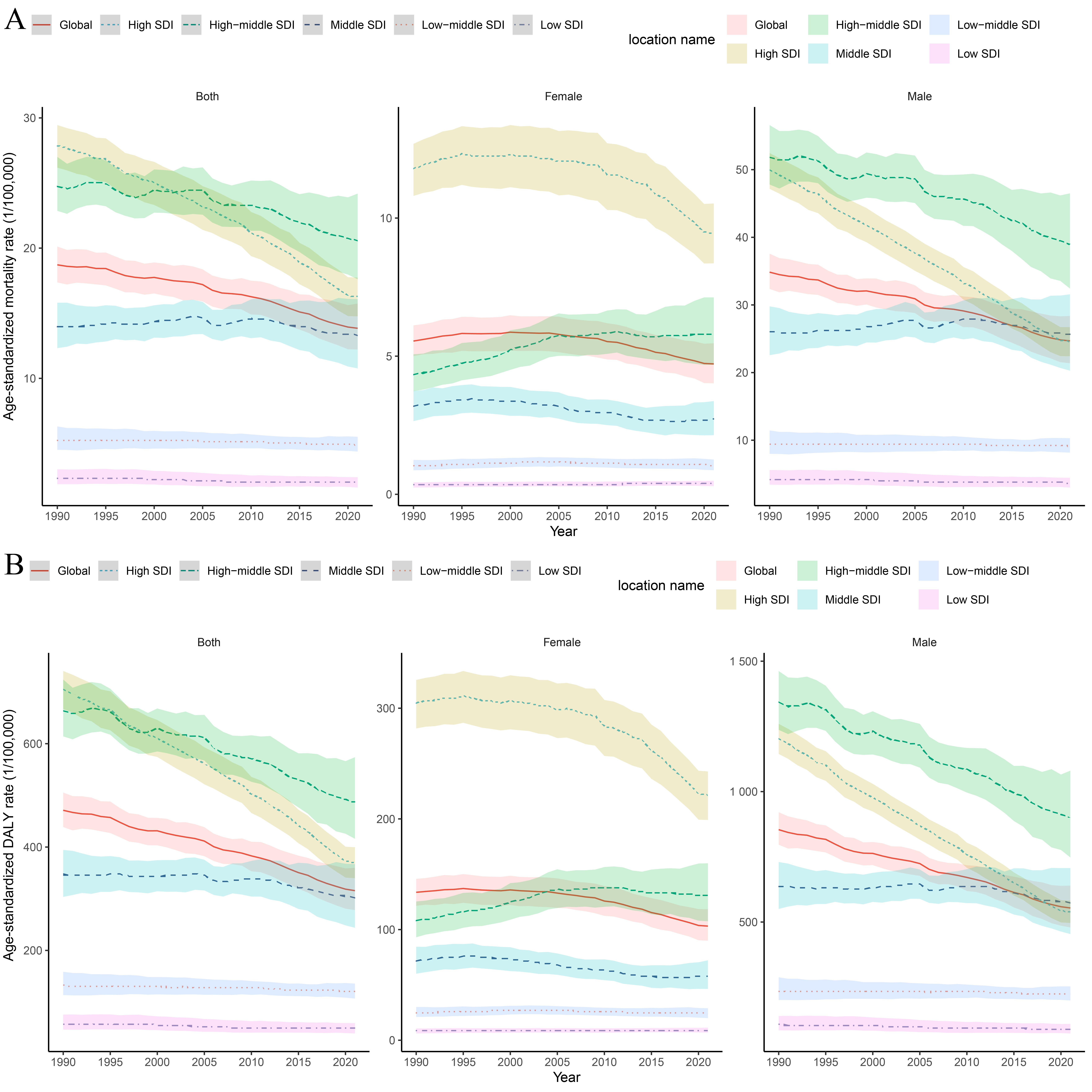

Supplement: Supplementary File 4. — Figure S2. Trends in age‑standardized mortality rate (ASMR) and age‑standardized disability-adjusted life years rate (ASDR) for tracheal, bronchus, and lung cancer globally by gender from 1990 to 2021. [file agh-90-1-4572-s4.tiff]
